# A critical appraisal of professional competency frameworks: What guidance is provided for stroke rehabilitation clinicians managing ‘complexity’?

**DOI:** 10.1177/26335565231215671

**Published:** 2023-11-18

**Authors:** Alyssa Indar, Michelle Nelson, Whitney Berta, Maria Mylopoulos

**Affiliations:** 1 Institute of Health Policy, Management and Evaluation, 7938 University of Toronto, Toronto, ON, Canada; 2 90755 Lunenfeld-Tanenbaum Research Institute/Sinai Health, Toronto, ON, Canada; 3 The Wilson Centre, 7938 University of Toronto and University Health Network, Toronto, ON, Canada

**Keywords:** professional competency frameworks, stroke rehabilitation, stroke distinction, complexity, critical appraisal

## Abstract

**Background:**

Given current health system trends, clinicians increasingly care for patients with complex care needs. There is a recognized lack of evidence to support clinician decision-making in these situations, as complex or multimorbid patients have been historically excluded from the types of research that inform clinical practice guidelines. However, expert clinicians at sites of excellence (e.g., Stroke Distinction sites) provide measurably excellent care. We sought to review profession-specific competency frameworks to locate information that may be supporting the development of clinician expertise when managing the care of patients with complex care needs.

**Methods:**

We conducted a review of the professional competency frameworks for core members of the inpatient stroke rehabilitation team, to determine the degree of guidance and/or preparation for the management of patients with complex care needs. We developed and applied an assessment rubric to locate references to patient complexity, multimorbidity and complexity theory.

**Results:**

Across the professional competency frameworks, there are some references to complexity at patient- and team-levels; there are fewer references to system-level complexity. We noted a lack of clear guidance for clinicians regarding the management of patients with complex care needs.

**Conclusion:**

Further research is needed to explore how clinicians develop expertise in the management of patients with complex care needs, as we noted minimal guidance in the professional competency frameworks. However, we suggest that integrating complexity-related language into professional competency frameworks could better prime novice clinicians for new learning in the workplace and ease their transition into working in a complex context.

## Introduction

Patients with multiple, concurrent health and social needs are frequently characterized as “complex” or as having “complex needs”.^1,2^ The care trajectories for patients with complex needs are often unpredictable and they require customized care that deviates from clinical practice guideline recommendations.^3-5^ This creates challenges for clinicians, who work within systems that are designed to align with standardized guidelines intended to support evidence-informed care delivery. In the absence of evidence that informs care for patients with complex needs, clinicians report needing additional time to problem solve or seek creative solutions.^2^ For new graduate or novice clinicians, managing care for patients with complex needs can be particularly challenging, while developing fundamental clinical skills.^6,7^

The learning curve for managing ‘complexity’ may be particularly significant in stroke rehabilitation settings, where clinicians report that it takes 12-18 months to feel competent or comfortable in their role.^8^ This raises the following questions: How do clinicians develop competence in managing the care of patients with complex needs? Particularly for new clinicians, what sources of knowledge do they draw on to guide their skill development in managing patient complexity? We present these questions to provide context regarding the motivation for developing this paper.

Stroke rehabilitation is an ideal setting to explore these questions for two reasons. Firstly, it is estimated that between 43-94% of patients recovering from a stoke have two or more co-morbidities, or qualify as multimorbid.^9^ Multimorbidity is linked to “medical complexity”; this is an important component of complexity, but using only this term may not clearly describe complexity arising from other sources, such as psychosocial, economic and health care utilization needs.^10^ Secondly, the process of stroke rehabilitation is highly structured. Within the Canadian context, the Canadian Stroke Best Practice Recommendations (CSBPR) are evidence-based guidelines that provide detailed practice expectations regarding the delivery of safe, high quality and patient-centred care.^11^ As suggested earlier, the guidance provided by the CSBPR may be limited in cases when clinicians are managing the care of complex patients. Research highlights the inappropriate fit between the guidance offered in clinical guidelines and the needs of patients with multimorbidity.^3,12^

When considering how stroke rehabilitation clinicians might learn to manage challenges related to patient complexity, we expect that there are a variety of learning inputs including formal education, practice-based training and continued “on-the-job” learning. A common thread amongst these inputs is their link to professional competency frameworks. Professional competency frameworks are distinct from practice guidelines in that they define the scope of practice and outline appropriate performance indicators for specific professions.^13^ Although the utility of competency frameworks is debated in the health professions education literature, reviewing these documents is a helpful starting point for understanding the professional expectations of clinicians.^13^ Competency frameworks are relevant to pre- and post-registration learning in clinical settings, as competency frameworks often inform performance expectations or indicators. It is reasonable to expect that there is language in competency frameworks that guides clinicians to develop an awareness or familiarity of complexity-related concepts, that in turn may support the development of foundational knowledge in managing patient complexity. Clinicians build on this foundational knowledge when they encounter in-practice opportunities to enhance their knowledge through experience and reflection. In this paper, we systematically review competency frameworks for all of the core inpatient stroke rehabilitation professions for references to managing “complexity”. Our understanding of the term ‘complexity’ was derived from seminal literature in complex adaptive systems theory, which describes complex systems as nested, interconnected, and co-evolving. Within these systems, agent actions may be characterized as unpredictable and non-linear, often leading to emergent and adaptive behaviours.^14-16^

Exploring profession-specific guidance for navigating problems that arise from the management of complex patient cases may help us to understand or map the foundational knowledge and skills on this topic. We acknowledge that our aim to search for ‘guidance’ (regarding complexity) may imply that we anticipate locating specific recommendations around complexity. However, this was not our expectation and not what we found. By ‘guidance’ we mean the presence of any complexity-related term, embedded in any professional competency statement. In turn, we contend that exposure to complexity-related language could support clinicians in developing an awareness of complexity, which could inform their management of patients with complex care needs.

The purpose of this paper is to provide a review of profession-specific competency frameworks, in order to identify complexity-related terms that may be supportive of the development of clinician expertise when managing the care of patients with complex care needs. When conducting our review within each professional competency framework, we posed the following questions:

(1) Are examples of complexity provided?
(2) Are specific clinician attributes described as helpful when managing complexity?
(3) Is explicit guidance provided for clinicians managing complexity?

## Methods

We conducted a critical review and appraisal within and across the competency frameworks (or documents) for clinicians working in inpatient stroke rehabilitation. Drawing on literature from the fields of patient complexity and multimorbidity,^1,17^ we developed and applied an assessment rubric which considered whether included articles: (1) provided examples of complexity, (2) specified clinician attributes (e.g., skills and behaviours) needed when managing complexity, and (3) offered explicit guidance for clinicians when managing complexity. When applying this assessment rubric, we used the broader term, “complexity”, as opposed to more specific terms such as “patient complexity” to increase the comprehensiveness of our review. The review consisted of three phases: (1) framework identification and retrieval, (2) application of the assessment rubric to qualitatively code the documents, and (3) synthesis of the data. The assessment rubric was customized to suit the purpose of this review. To design and apply the rubric we relied on methods similar to those used by Nelson and colleagues.^5^

### Phase 1: Framework identification and retrieval

As stated earlier, the CSBPR describes resources, processes, and structures necessary to deliver safe and high-quality care in stroke rehabilitation settings. This includes a clear description of the “core” and “additional” members of the patient care team in the CSBPR.^11^

The following professions were specified as core members of the health team: *physiatrists,* or *other physicians with expertise/core training in stroke rehabilitation, occupational therapists, physiotherapists, speech-language pathologists, nurses, social workers* and *dietitians [Evidence Level A].* The *patient* and *family* are also included as part of the core team *[Evidence Level C].* The following professions were characterized as additional team members: *recreation therapists, psychologists, vocational therapists, educational therapists, kinesiologists, rehabilitation therapy assistants,* and *pharmacists*.^11^

For the purposes of this review, we selected competency frameworks that corresponded to each of the “core” team members. We located the professional competency frameworks by downloading them from the provincial regulatory website of each profession. Table 1 provides a list of the competency frameworks that were retrieved for each of the listed professions.

**Table 1.** List of Selected Professional Competency Frameworks.

Profession	Competency Framework
Physiatrist	Royal College of Physicians and Surgeons of Canada [RCPSC]: Physical Medicine and Rehabilitation Competencies (effective for residents after July 1, 2020)^18^
Registered Nurse (RN)	College of Nurses of Ontario [CNO]: Entry-to-practice Competencies (revised December 2018; to take effect September 2020)^19^
Registered Practical Nurse (RPN)	College of Nurses of Ontario [CNO]: Entry-to-practice Competencies (revised April 2019; to take effect September 2020)^20^
Physiotherapist (PT)	College of Physiotherapists of Ontario [CPO]: Physiotherapist Essential Competencies (2017)^21^
Occupational therapist (OT)	College of Occupational Therapists of Ontario [COTO]: Essential Competencies of Practice for Occupational Therapists in Canada (3^rd^ edition, May 2011)^22^
Social worker (SW)	Ontario College of Social Workers and Social Service Workers [OCSWSSW]: Code of Ethics and Standards of Practice Handbook (2^nd^ ed, 2008; Includes amendments effective October 1, 2010, February 13, 2011, May 6, 2015, January 1, 2018 and September 7, 2018)^23^
Speech language pathologist (SLP)	College of Audiologists and Speech-Language Pathologists of Ontario [CASLPO]: National Speech-Language Pathology Competency Profile (May 29, 2018)^24^
Dietician	Partnership for Dietetic Education and Practice [PDEP]: Integrated Competencies for Dietetic Education and Practice (Version 3.0 (July 6, 2020)^25^

### Phase 2: Assessment rubric application

Professional competency frameworks are intended to be comprehensive and applicable to practitioners working in a variety of clinical settings. Given the wide scope of these documents, creating an assessment rubric to guide the document review was an essential step in focusing the qualitative coding process. The assessment rubric was co-developed with MN, using methodology similar to previous work which critically appraised the Stroke Rehabilitation Best Practice Recommendations.^5^ As stated earlier, we used the broad term “complexity” in the assessment rubric. The rationale for this decision is rooted in our understanding of applications of complexity theory within the domain of health care. For example, prior theoretical work has described health care systems and organizations as complex adaptive systems^26^ thus, underscoring the interconnected nature of care delivery.^27-29^ Therefore, we anticipate that concerns about managing patient-level complexity could manifest as “complex problems” at the team, organizational, or system level.^16^

Below are the three main assessment questions, supported by sub-questions:

(1) Are examples of complexity provided?

• What terms are commonly used to characterize complexity?
• What type(s) of complexity are often referred to? For example, is complexity described at patient, clinician, team, organizational or system levels?

(2) Are specific clinician attributes (e.g., skills, behaviours, attitudes) described as helpful when managing complexity?

• If so, what attributes, skills, behaviours or attitudes are described?

(3) Is explicit guidance provided for clinicians managing complexity?

• If so, what level of guidance is provided and what are the key practice recommendations?

The assessment rubric draws upon literature on the topics of patient complexity, multimorbidity and complexity theory, which is described further in the first author’s dissertation (Indar, 2023).^30^ In Table 2, we list examples of keywords that correspond to these topics. The keywords correspond to major concepts in the selected literature. Through team discussion, we agreed on which keywords would be most relevant for inclusion in the assessment rubric. Selecting specific keywords provided further direction for the coding process, in which we searched for these or similar keywords in the competency documents. To improve transparency, we list one seminal reference that corresponds to each topic in the table; however, the comprehensive literature review is included in the dissertation (Indar, 2023).^30^

**Table 2.** List of Keywords Informing the Appraisal Framework.

Topic	Keyword Examples
Patient complexity^1^	Complex health needs Health and social needs Social/psychological/economic/spiritual needs
Multimorbidity^17^	Multiple co-morbidities Medical needs
Complexity theory^14-16^	Complex Unpredictable Non-linear Interconnected/embedded Emergent Dynamic Co-evolving/learning Adaptive/adaptation

### Phase 3: Data analysis

When reviewing each professional competency framework, the first author (AI) searched for references to the keywords in Table 2. When competency statements with these keywords were identified, they were copied to a Word document. The Word document was sorted by profession, to facilitate later comparison across professions. Data were organized purposefully to group similar references together. This document was reviewed throughout the data analysis process and shared at regular intervals with the co-authors, to inform analytic discussion. The document contained the competency statement excerpts, or data, and was coded; similar competency statements were grouped together to build categories. This process was completed in accordance with the principles of content analysis.^31,32^

After reflecting on the data and constructed categories, our team decided to present the data in a way that highlighted the emergent patterns. For example, the questions guiding the review helped us to locate important pieces of information (e.g., examples of complexity, clinician attributes when managing complexity, and examples of clear guidance). However, we noticed patterns during the data analysis process, corresponding to types of complexity in practice: (1) at the person/patient level, (2) the team level, and the (3) system level. Given the varied conceptualizations of the ‘complexity’ in practice, we thought it helpful to organize the results using these headings.

## Results

We observed differences in the structure and organization of the reviewed competency frameworks. Most competency frameworks outlined practice expectations for professions that were applicable to all clinical settings and applied across Canada. An exception was the nursing profession, as the corresponding frameworks were specific to Ontario. Documents were organized in different formats, such as roles,^18-20,24^ workplace abilities,^25^ or principles.^23^ These formats offered different levels of specificity in the recommendations for clinicians. This section provides an overview of the detailed findings, organized into categories that correspond to the assessment rubric.

### Section 1: Are examples of complexity provided?

In this section, references to complexity and/or proxy terms are organized by patient-, team- or system- level.

#### Person and/or Patient

All competency frameworks mentioned either *patient-centred care* or *client-centred care*, which indicated a clear expectation that the needs and preferences of patients and families be considered in the rehabilitation process. In most competency frameworks, there were references to different patient dimensions or domains representing physical, social, psychological, and economic needs. Competencies for physiatrists were identified as they related to all of these domains but emphasized physical aspects by using terms such as ‘complex medical conditions’, and ‘multiple medical comorbidities’. The OT competencies expanded on the need to develop ‘experiential knowledge of the client’ and integrate knowledge of client values and meanings related to their goals into care.^22^ Similarly, RN and RPN competencies described a need for ‘holistic assessment’ and explicitly acknowledged the spiritual domain.^19,20^

There were some differences in understanding the barriers to care for patients. Some competency frameworks emphasized recommendations to mitigate challenges at the level of the individual patient (e.g., communication barriers). Notably, many of the competency frameworks integrated a component of advocacy and emphasized the need to address system level barriers that impede patient access to care due to psychosocial or economic constraints.^18,19^

In summary, our review elucidated how ‘complexity’ is described in relation to the concept of patient-centred care. There is general recognition of the need to view patients holistically, but we found there remains an emphasis on medical or physical management. There were clear directives for clinicians to acknowledge the experiences of patients and barriers to care at the individual and system levels. Tables 3, 4, 5, 6

**Table 3.** Patient-level References to Complexity.

	MD	OT	PT	SLP	OT	RN	RPN	Diet
**Terms for Complexity**								
-Patient-centred care	X							
-Client-centred care		X	X	X	X	X	X	X
-Holistic care						X	X	
**Terms for Multimorbidity**								
-Co-morbidities	X		X					
-Complex medical conditions	X

**Table 4.** Team-level References to Complexity.

	MD	OT	PT	SLP	RN	RPN	SW	Diet
**Terms for Team**								
-Interprofessional team	X	X	X		X	X		X
-Multidisciplinary team							X	
-Teamwork (and related terms)	X	X	X					X
-Collaboration (and related terms)	X	X	X	X	X	X	X	X

**Table 5.** System-level References to Complexity.

	MD	OT	PT	SLP	RN	RPN	SW	Diet
**Explicit reference to ‘complexity’ in practice**	X							
**Patient safety**								
-Macro-level language (e.g., culture)	X		X					X
-Micro-level language (e.g., reports errors)	X	X	X	X	X	X		X
**Engagement in QI**								
-Macro-level language (e.g., planning)	X		X					
-Micro-level language (e.g., implementing)	X	X	X	X	X	X		
**Resource Management/Allocation**								
-Macro-level language (unit/org level)	X	X	X					X
-Micro-level language (individual practice level)	X	X		X	X	X		X

**Table 6.** Clinician Skills Related to Functioning in Complex Contexts.

	MD	OT	PT	SLP	RN	RPN	SW	Diet
**Resource Management**								
-Need skills to manage multiple demands	X	X	X	X	X	X		X
**Learning**								
-Learning via reflection	X	X	X	X	X	X		X
-Participates in mentorship (different from teaching)		X		X		X		
**Clinician Well-being**								
-Ability to manage personal/professional demands	X	X	X		X			

.

#### Team

Working effectively in a team-based clinical environment is a complex process, particularly when collectively managing care for patients with complex needs.^33^ In all competency frameworks, working as a part of a health team was mentioned, using a variety of terms: ‘interprofessional team’, ‘teamwork’, ‘team dynamics’, ‘collaboration’, ‘collaborative practice’, ‘collaborative care’, and ‘relationship-centred collaborative care’. In competency frameworks that were organized by roles, the role of ‘collaborator’ included content on teamwork principles related to effective communication, role clarity and conflict resolution.^18,19,24^ In most competency frameworks, the principles of teamwork were well described and appeared to underpin professional practice. This suggests that teamwork or collaborative practice is a core competency for clinicians.

The descriptions of the team also provided insight into the specific roles of individual clinicians within the team. For example, the physiatrist competencies described the need to work with and within different teams in different rehabilitation settings (e.g., inpatient, outpatient). Descriptions of professional relationships frequently differentiated between interactions with physician and non-physician colleagues, as indicated by the following example of phrasing: “physicians and other colleagues in health care professions”.^18(p13)^ This is in contrast with competency statements from other professions that referred to the generic term ‘team member’ to describe a range of health professions. The teamwork-related competencies for RNs were primarily linked to the communicator, collaborator and coordinator roles, which identified common nursing actions to communicate, consult and inform team members about changes in client status or care.^19^ The RPN competencies were similar in nature, although not linked to a particular role.^20^ Both RN and RPN competency frameworks implied that nurses deliver care within the context of a team.^19,20^ The PT competency framework outlined a requirement to first “identify practice situations that may benefit from collaborative care”^21(p12)^ and follow up with additional competency statements that provide expectations for teamwork, in similar fashion to the OT and dietician competencies.^22,25^ There was a lack of guidance for teamwork in the SW competencies, which could reflect the diversity of roles for SWs in health and social services.^23^ Overall, there were clear teamwork-oriented expectations for most clinicians in their corresponding competency frameworks. The complex nature of working within teams was referenced in most competency frameworks including one or more statements providing guidance on managing team communication or conflict.

#### System and/or Process

There were few references to system level complexity in the competency frameworks. In the physiatry competencies, there was one direct reference: “Recognize and respond to the *complexity, uncertainty, and ambiguity* inherent in practice”.^18(p6)^ This statement seemed to align directly with descriptions of complex adaptive systems, as they are characterized by uncertainty, unpredictability, and non-linearity.^16^ The OT competencies alluded to the layered complexity of the health care environment by setting the expectation that the clinicians be aware of the “physical, social, cultural, institutional and economic environment relevant to the jurisdiction of practice”.^22(p12)^

Systems-level thinking was reflected in most competency statements that addressed patient safety. As expected, patient safety was embedded in all requirements for clinicians and the language of the competency statements emphasized that clinicians should understand patient safety in relation to the larger health system, rather than at the individual level. For example, the following OT competency statement referenced health systems: “Shows awareness of *health systems*, error, and client safety concepts”.^22(p32)^ This was also reflected in specific expectations for clinicians to “contribute to organizational culture of safety”,^25(p22)^ and “analyze patient safety incidents to enhance systems of care”.^18(p14)^ Further acknowledgement of systems-level thinking was observed in competency statements that described an expectation for clinicians to understand the importance of engaging in and contributing to quality improvement projects. This suggested that clinicians should be engaged in methods to improve not only personal practice, but team or organizational level practice changes.

Few competency frameworks described an expectation that clinicians consider or contribute to appropriate resource allocation.^18^ Understanding resource management was a key competency listed under any roles related to leadership, particularly for physiatrists. This could be reflective of the default macro-view of the health care system held by physiatrists, due to the requirement of their role to work frequently across teams and organizations.

### Section 2: Are specific clinician attributes (e.g., skills, behaviours, attitudes) described as helpful when managing complexity?

Given that there were minimal explicit references to ‘complexity’, it was difficult to ascertain which described skills or attitudes could be useful when encountering a complex patient or case. To address this assessment rubric question, we grouped together any skills that seemed related to working within unpredictable or dynamic contexts. There were a few competency statements that directed clinicians to recognize when a practice situation becomes complex and adapt their thinking or actions accordingly. Below are three selected examples:

• Physiatry: “Recognize *practice uncertainty* and knowledge gaps in clinical and other professional encounters and generate focused questions that can address them”^18(p18)^
• OT: “Demonstrates *situational awareness* by continually observing the whole environment, thinking ahead, and reviewing potential options and consequences”^22(p28)^
• RN: “Communicates effectively in *complex and rapidly changing situation*”^19(p6)^

From these and similar competency statements, three main skills were identified that support clinician management of a “complex” clinical situation. The first skill is the need to effectively manage time and resources within individual practice. Many competency statements described the practice environment as creating competing demands, in which a clinician develops clinical expertise to navigate it appropriately. The second skill is the need to continuously learn from practice. All competency frameworks referenced the need to learn from evidence; for example: “Adapts to changes in practice using evidence, practice standards, and best practices”.^22(p29)^ Most referenced the need to reflect on practice, where a variety of terms reflected different depths of reflection: ‘self-evaluation’, ‘self-reflection’, ‘reflective practice’, and ‘critical inquiry’. The third skill was related to balancing professional and personal demands in a way that protected or enhanced clinician well-being. These skills featured prominently in the physiatrist, PT and OT competencies, for example:

• “Manage personal and professional demands for a sustainable practice throughout the physician life cycle”^18(p20)^
• “Promote a culture that recognizes, supports, and responds effectively to colleagues in need”^18(p20)^
• “Maintain personal wellness consistent with the needs of practice”^21(p19)^
• “Manages professional responsibilities by recognizing personal and professional limits of functioning”^22(p29)^

These statements reflect the importance of individual clinician well-being and attending to workforce well-being to optimize patient care. These skills were described in most detail as they related to the physiatrist competencies, where clear expectations for caring for self and for colleagues were delineated. There were few competency statements related to self-care in the nursing and social work documents.

### Section 3: Is explicit guidance provided for clinicians managing complexity?

The findings from this appraisal show that there is very little explicit guidance provided for stroke rehabilitation clinicians managing complexity at patient-, team-, or system-levels. Many statements require clinicians to demonstrate an ‘awareness’ of the concepts related to complexity, such as complex patient needs or organizational cultures of safety, without providing clear direction in terms of how awareness is achieved and reflected in particular skills, behaviours or actions. Greater specificity could: (1) provide enhanced understanding and guidance for specific clinical actions in complex situations, and (2) provide a stronger foundation to start formally building considerations of complexity into professional practice and academic programs.

### Summary

In searching for complexity-informed language at the patient-, team-, and system-levels, there were a few notable findings. As mentioned earlier, the expectation that clinicians practice in a patient or client-centred way is clearly stated. Although different professions appear to focus on different dimensions of complexity, relative to their role and relationship with the patient, there appears to be a growing awareness of the broad range of barriers that patients encounter while receiving care.

There is agreement regarding the importance of teamwork, indicated by the inclusion of collaborative principles across competency frameworks. This signals a broad recognition that health care delivery is primarily team-based. There was much variation at the system-level regarding the awareness of macro-level factors that could contribute to complexity. For example, most competencies covered concepts related to patient safety, quality improvement and resource management or allocation. The professions that are typically perceived as those with leadership roles, such as physiatry, were required to understand these concepts at a system level. In slight contrast, professions less likely to occupy formal leadership roles (e.g., nursing) were expected to understand these concepts within the context of individual practice. Table 7 provides a visual summary, which highlights how the results correspond with the guiding review questions.

**Table 7.** Summary of Assessment Questions and Key Results.

Assessment Questions	Key Results
(1) Are examples of complexity provided?	• All competency frameworks mentioned either *patient-* or *client-centred care*; although there were more references to managing the physical aspects of care (as opposed to psychosocial, etc.) • Most competency frameworks had strong references to *teamwork* and *collaboration.* • There were few references to system level complexity in competency frameworks.
(2) Are specific clinician attributes described as helpful when managing complexity?	There were a few competency statements that directed clinicians to recognize when a practice situation becomes complex and adapt their thinking or actions accordingly. For example: • MD: “Recognize practice uncertainty and knowledge gaps in clinical and other professional encounters and generate focused questions that can address them” • OT: “Demonstrates situational awareness by continually observing the whole environment, thinking ahead, and reviewing potential options and consequences” • RN: “Communicates effectively in complex and rapidly changing situation”
(3) Is explicit guidance provided for clinicians when managing complexity?	Many statements require clinicians to demonstrate an ‘awareness’ of the concepts related to complexity, such as complex patient needs or organizational cultures of safety, without providing clear direction in terms of how awareness is achieved and reflected in particular skills, behaviours or actions.

Detecting skills or attitudes important for clinicians working in complexity was challenging and required our team to make inferences. Descriptions of complexity were vague and required the reviewer to locate proxy terms that aligned with concepts referenced in complexity theory or describe the attributes of a complex adaptive system.^16^ Terms such as ‘uncertainty’ or ‘rapidly changing’ were identified and linked to key skills related to methods of learning, resource management and clinician wellness. It was particularly difficult to locate required attitudes of clinicians, beyond the demonstration of a commitment to patient-centred or high-quality care. Overall, this review highlighted two main areas where more specific information could be helpful for clinicians. More description is needed to understand: (1) how practice complexity is experienced and interpreted by clinicians, and (2) what skills and attitudes are required to manage practice complexity, stemming from a range of sources (such as patient, team, system). These descriptions could provide a foundation for developing explicit guidance for clinicians managing complexity.

## Discussion

In this focused appraisal of professional competency frameworks for stroke rehabilitation clinicians, we systematically searched for references to managing “complexity”. We explored the implications of our two main findings in this section.

The first key finding from this review is that the use of the specific term “complexity” is limited, but there is a clear presence of proxy terms throughout the competency frameworks. We were able to locate these proxy terms, as our appraisal framework supported a search for terms related to complexity theory - such as “uncertain”, “unpredictable”, “dynamic” or similar. These terms referenced different sources and manifestations of complexity, such as patients with complex needs, complex team dynamics, complex systems, complex problems, complex cases, complex clinical contexts, complex workplace processes and other related concepts. Essentially, there were multiple aspects of clinical practice that could be considered “complex”, which hindered our efforts to clearly define the sources of “complexity” in the stroke rehabilitation setting and how they are experienced by clinicians. The lack of clarity and consistency regarding terms related to “complexity” makes the formulation of explicit and concise guidance particularly difficult. Expectedly, this is challenging for clinicians learning to recognize a complex patient, case or problem. The absence of clarity regarding “complexity” suggests that research informing a conceptual definition might be useful and could inform future iterations of competency frameworks.

The second key finding from this review includes a few skills that may be valuable for clinicians working in complex clinical environments, characterized by unpredictability and uncertainty. These skillsets include adaptability, self-reflection, and self-care. The concept of ‘adaptation’ features strongly in literature related to complexity theory, as health care organizations and teams have been characterized as “complex adaptive systems”.^27,34^ In addition to describing health care systems and environments as adaptive, it appears that clinicians working in these systems also need a flexible and adaptive mindset to quickly and competently adjust to changes related to their patients’ health statuses and work environments (e.g., team ways of working, organizational policies and procedures). There may also be value in exploring ways in which organizations can support adaptive practices.

Clinician engagement in self-reflection may also support skills in adaptation, as reflective practice promotes critical thinking and learning that enhances clinician competence.^35^ Clinician development of adaptive and reflective practices are considered high-level skills, cultivated through experience and purposeful learning. For example, clinicians in stroke rehabilitation report that it takes up to 18 months to feel competent in managing complexity, which suggests that they endure a prolonged and intense period of learning.^8^ This underscores the importance of self-care for clinicians learning to manage complexity in stroke rehabilitation and other similarly challenging contexts. As highlighted in this review, references to self-care were found most frequently in competency frameworks for physiatrists, physiotherapists and occupational therapists.^18,21-22^ Although heretofore un-emphasized for other professions, self-care for social workers and nurses, who are known to experience significant emotional labour by virtue of the ‘helping’ nature of their roles, is likely important.^36^ Discussions about the concept of self-care for clinicians appears to be gaining traction, possibly due to the increasing complexity of patients across care settings and the resource-constrained health care system contexts.

Although our review confirmed that phrasing of competency statements acknowledged the complexity of clinical practice, a third key finding is that competency frameworks do not provide easily identifiable and explicit guidance for stroke rehabilitation clinicians seeking support for managing complexity. The issue of supporting clinicians in navigating the complex aspects of clinical practice is not unique to stroke rehabilitation, as patients with complex needs require care across the healthcare continuum. Recent work by Batt and colleagues^37^ underscores the need to use systems thinking, including complexity theory, to inform the development of professional competency frameworks. Similar research suggests that creating professional competencies to support systems-based practice could improve our collective ability to address structural problems, such as health inequities.^38^

Competency frameworks may be providing cues to support clinicians in recognizing when they are encountering a complex situation (e.g., uncertain, unpredictable, etc.) and stimulate a shift into a complex problem-solving mode. For example, the Cynefin framework is an example of a decision-making framework and applications in complex contexts suggest that clinicians employ a “probe-sense-respond” approach, in which they look for patterns and co-develop an emergent solution within their multidisciplinary care team.^39^ Key to enacting this approach is leveraging teamwork to develop creative solutions. Similar research suggests that clinicians rely on mindlines or “guidelines-in-the-head”, which are informed by a clinician’s “training, their own and each other’s experience, their interactions with colleagues and patients, by their reading, their understanding of local circumstances and systems, their experiences of handling the many conflicting demands, and a host of other influences.”^40(p402)^ This work also emphasizes the role of teamwork, since a critical component of building mindlines is interaction with others, including interprofessional team members. For teams that routinely manage care for a large proportion of complex patients, as is the case in stroke rehabilitation contexts, engagement in generative discussion could support the search for innovative solutions. To date, research theorizing how clinicians manage complex cases has highlighted the importance of relationship-building and collaborative learning.^40^ Further research might focus on extending our understanding of how clinicians recognize and collectively manage the care of patients with complex needs, particularly within highly structured contexts, such as stroke rehabilitation. Such work could clarify how teamwork may differ when caring for patients with particularly complex care needs, and therefore suggest how this mode of ‘teamworking’ could be better supported in education and practice settings. Further research may also be helpful to better explore the initial questions posed in the background section, regarding: (1) how clinicians develop competence in managing the care of patients with complex needs, and (2) what sources of knowledge clinicians draw on to guide their skill development in the management of patients with complex needs.

## Limitations

In this paper, we reviewed the professional competency frameworks for core members of inpatient stroke rehabilitation teams, to determine the degree of support for the management of patients with complex care needs. To accomplish this, we developed and applied an assessment rubric to locate terms that were indicative or suggestive of the complexity that clinicians confronted in stroke rehabilitation contexts. During the process, a potential limitation was the degree to which all team members engaged in the analytic activities. As this comprised a portion of the first author’s PhD dissertation, she led the application of the assessment rubric. To minimize bias, co-authors were engaged in analytic discussions at regular intervals. At these meetings, the co-authors reviewed the data and posed critical questions related to the study methods and emerging insights. The varied perspectives and expertise of the co-authors contributed to rich analytic discussion that have enhanced this review.

## Conclusions

Stroke rehabilitation clinicians routinely provide team-based care to a high proportion of patients with complex care needs. In this paper, we reviewed competency frameworks for all core stroke rehabilitation professions for references to managing “complexity”. Exploring profession-specific guidance for navigating problems that arise from the management of complex patient cases may help us to understand or map the foundational knowledge and skills on this topic. We learned that complexity has many proxy terms and that clinicians encounter different types of complexity that may in large part stem from complex patient needs, complex team dynamics, and system-level complexity. For clinicians, the intersecting sources of complexity may simply present as a “complex problem” and their collective priority is to manage it in a way that satisfies patient needs, while adhering to system constraints. Prior research indicates that clinicians may achieve this by leveraging the expertise of their team to seek emergent and creative solutions to novel problems.^39,41^ Although current competency frameworks afford limited guidance for the management of complex problems, they could play an important role in supporting clinicians to recognize complexity, which could in turn cue them to seek collaborative solutions. Further research that clarifies how clinicians recognize and manage complexity could inform future iterations of professional competency frameworks and other documents that provide guidance for clinicians. This is particularly important for clinicians that routinely encounter complexity in their practices, such as those working in stroke rehabilitation.
